# Microstructure inhomogeneity of Fe-31%Ni alloy and stabilization of austenite

**DOI:** 10.1186/s11671-015-0785-7

**Published:** 2015-03-11

**Authors:** Ievgenij M Dzevin

**Affiliations:** G.V.Kurdyumov Institute of Metal Physics NAS of Ukraine, Vernadsky blvd. 36, Kiev, 03680 Ukraine

**Keywords:** Melt-spun ribbon, Microstructure, Austenite, Martensitic transformation, X-ray diffraction, 68.35.bd Metals and alloys, 68.35.Rh Phase transitions and critical phenomena, 68.37.-d Microscopy of surfaces, interfaces, and thin films

## Abstract

Сrystal structure and mechanism of crystallization of Fe-Ni alloys were studied by methods of X-ray diffraction and metallography. It has been found that macro- and microstructure of austenitic alloy was essentially heterogeneous at the contact and free surfaces and in the volume of a ribbon. The indentified peculiarities of the austenitic phase in different areas of the ribbon are attributed to different cooling rates and the melt crystallization conditions.

## Background

Crystallization of metals from the melt occurs under sharply non-equilibrium conditions and resulted from ultra-fast cooling, different heat abducting gradient on different surfaces of melt-spun ribbon formed, possible redistribution of alloying elements, and the formation of the specific structural-and-stress state. These factors can cause a significant macro- and microinhomogeneity of thin melt-spun ribbons. During the phase transformation in thin ribbons of metastable alloys, these inhomogeneities can lead to considerable changes of characteristics of the transformations and, therefore, can cause a change of physical and mechanical properties in the local areas of melt-spun ribbon.

In particular, in the Fe-Ni alloys, given high temperature and concentration gradients during quenching from melt, the characteristics of martensitic transformations are used to be determined by the cooling speed and the redistribution of nickel on different surfaces of the ribbon and also by the dimension effect of the transformation [1-4].

The purpose of this work is to study the principles of distribution of martensitic phase in the local areas of metastable Fe-31 wt.% Ni melt-spun ribbons with heterogeneous microstructure formed during quenching from melt.

## Methods

A metastable alloy Fe-30.6 wt.% Ni-0.05 wt.% С chosen as an object of investigation was prepared by induction melting. This alloy in the initial state at a room temperature was in the austenitic state. Fast-quenched alloy prepared as thin ribbon (thickness of 40 to 50 μm and width of 8 mm) was fabricated in the carbon dioxide environment at 10^5^ to 10^6^ K/s cooling speed by melt spinning method from 100 g of alloy [5]. Temperature range of the direct and the reverse martensitic transformations was defined by a differential magnetometer. The magnetic field shown by the magnetometer was 10 kOe, the temperature was measured in the range of 77 to 773 K, and the amount of martensitic phase was measured with the accuracy of 0.5%. The temperature points of the investigated alloy were *M*_s_ = 213 K, *M*_f_ = 153 K, *A*_s_ = 573 K, and *A*_f_ = 653 K. Direct γ-α transformation in ribbons and bulk specimen realizes by cooling in liquid nitrogen. The reverse α-γ transformation with diffusionless character realizes by heating in salt bath with rate 60 K/s. The reverse α-γ transformation with diffusional character in the ribbon was realized on a slow heating at a speed of 1.33 × 10^−2^ K/s in the atmosphere of argon up to the temperature 823 K. Structure and phase analysis of ribbons was performed by X-ray diffraction method with the DRON-3 diffractometer at a room temperature. Amount of martensitic phase was determined by comparing of intensity of the (111)_γ_ and (110)_α_ diffraction reflexes. Microstructure investigations of ribbons were carried out by means of PREM-200 electron microscope and NeoPhot optical microscope.

## Results and discussion

Dimensional effect of the direct martensitic transformation can have an influence on the reversed transformation. In our study, the influence of the reversed transformation in microcrystalline fast-quenched alloys on completeness of the next direct transformation and on the stabilization of reversed austenite has been investigated. Completeness of direct γ-α transformation in the ribbon is mainly estimated by amount of ultradispersed and nanocrystalline components of grains, where γ-α transformation was partly or fully suppressed. Indeed, it was shown earlier that martensitic crystals could not form in nanocrystalline grains of bulk Fe-Mn-C and Fe-Ni alloys [6,7]. Amount of martensite decreases in dispersed graines and austenite twins [8].

The reverse α-γ transformation in the ribbon was realized on a slow heating at a speed of 1.33 × 10^−2^ K/s in the atmosphere of argon up to the temperature 823 K. On these conditions, the α-γ transformation have the diffusional character and therefore, multiplying of γ-phase crystallographical orientations occur according to orientation relationships between the crystalline lattices of austenite and martensite. The differently oriented nanosize (50 to 100 nm) crystals of reverted austenite in the form of the plates are forming according to γ-phase orientations multiplication [3,9,10]. After the first γ-α-γ transformation cycle with diffusive character of atomic redistribution, the sizes of austenitic grains appeared much smaller than the critical size. Hence, reverted austenite was fully stable and the next quenching in liquid nitrogen yielded full suppression of γ-α transformation resulted from the dimensional effect.

Furthemore, the characteristics of martensitic transformation were investigated in a bulk alloy used for further preparation of melt-spun ribbons. After the first cycle of the γ-α-γ transformation, the cooling in liquid nitrogen appeared to result in formation of 26 vol.% of martensite; after the second cycle, it resulted in 8 vol.% of martensite. After the third cycle of transformation, reverted austenite was fully stabilized. These results showed that a degree of austenite fragmentation caused by the reverse transformation in the ribbon had been considerably higher than in the bulk alloy of the same chemical composition.

**Figure 1:**
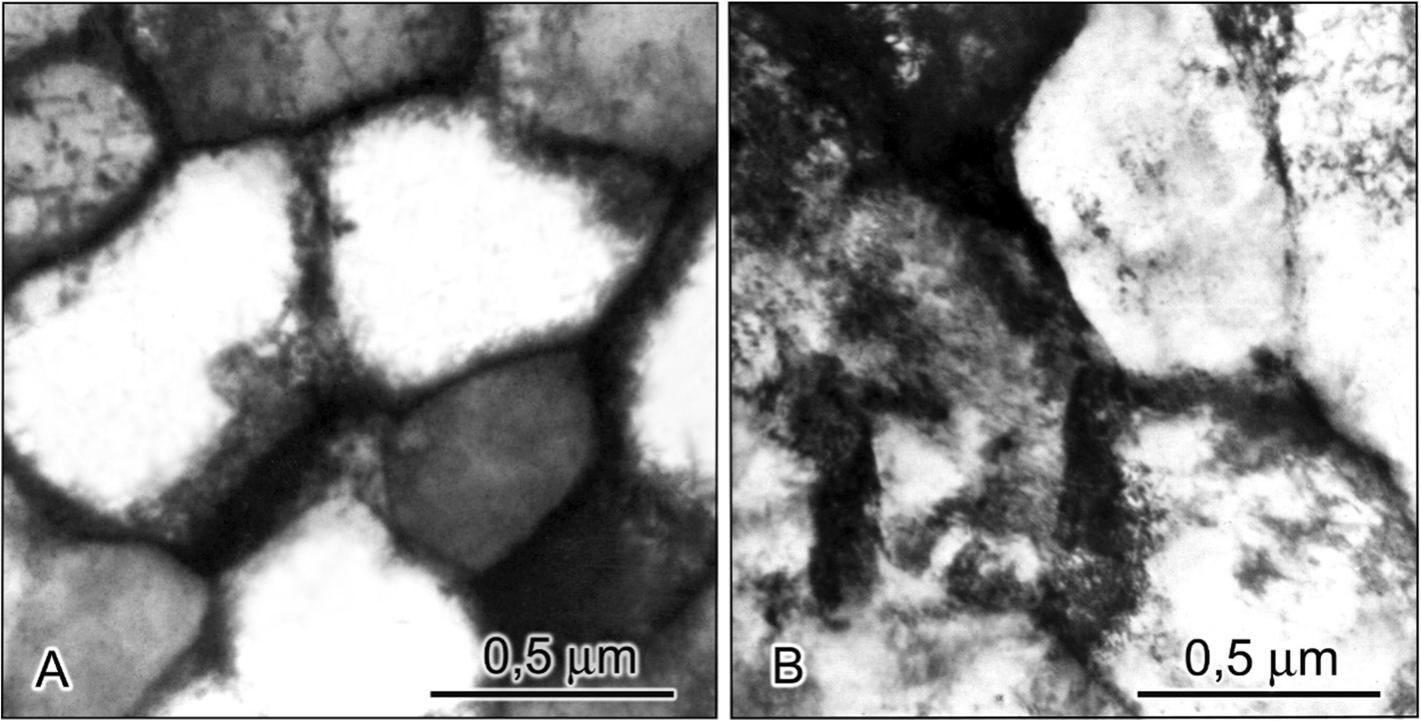
**Microstructure of austenite in initial state (A) and after repeated γ-** **α-** **γ transformations (B) of melt-spun ribbon of Fe-30.6 wt.% Ni-0.05 wt.% C alloy.** ×20,000. Reverse transformation that occurred on heating of quenched alloy in the temperature interval of α-γ transformation at a speed higher than critical one has a diffusionless character. Under this character of atomic redistribution, the degree of stabilization of the austenite reverted by γ-α-γ transformations appeared considerably lower than in the case of the diffusive character of reverse transformation. It happened since diffusionless reverse transformation did not change the size of initial austenitic grain but the boundaries of grain become unclear and distorted. Certain substructure shows up in a volume of grain. After tens of diffusionless α-γ transformations, the fragments of different orientation of reverted austenite reached nanoscale size.

**Figure 2:**
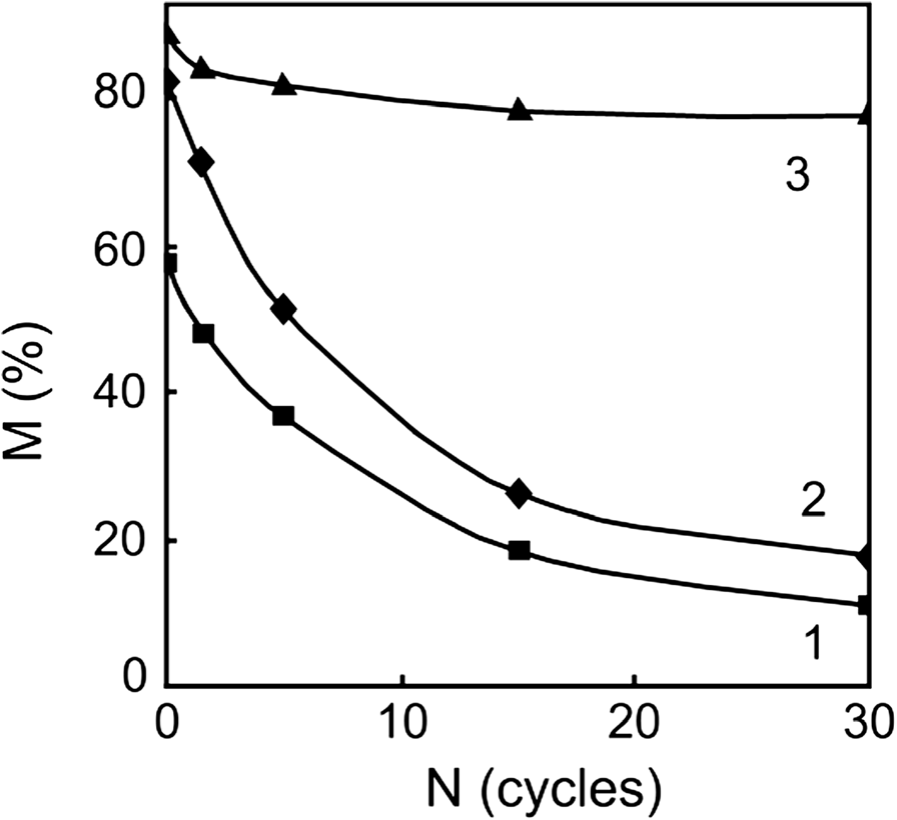
**Martensitic volume part vs the number of γ-** **α-** **γ cycles.** Change of the martensitic volume part vs the number of γ-α-γ cycles for the contact (1), free (2) surfaces of a spinning ribbon, and bulk (3) Fe-30.6 wt.% Ni-0.05 wt.% C alloy. Reduction of the size of austenitic grain in melt-spun ribbons results in more considerable stabilization of reverted austenite as compared to the bulk alloy of the same chemical composition. It was found that after 30 γ-α-γ cycles of reverse diffussionless transformations in the ribbon, the amount of martensite on a free side dropped by 61%, on a contact side by 43%, while in a bulk alloy, only by 8%. Investigations indicated that the size effect of martensitic transformation yielded a higher degree of stabilization of reverted austenite on a free side of the ribbon as compared to a contact side. Quenching in liquid nitrogen caused essential difference of grain structure on both the sides of the ribbon as well as the gradient distribution of the amount of martensitic phase inside it. Austenitic grains on a contact surface was smaller than on a free surface. As a result of the size effect of the transformation, amounts of martensitic phase on a contact and free surfaces were different - 59% and 82%, respectively.

Reverse transformation that occurred on heating of quenched alloy in the temperature interval of α-γ transformation at a speed higher than critical one has a diffusionless character. Under this character of atomic redistribution, the degree of stabilization of the austenite reverted by γ-α-γ transformations appeared considerably lower than in the case of the diffusive character of reverse transformation. It happened since diffusionless reverse transformation did not change the size of initial austenitic grain but the boundaries of grain become unclear and distorted (Figure 1B). Certain substructure shows up in a volume of grain. After tens of diffusionless α-γ transformations, the fragments of different orientation of reverted austenite reached nanoscale size [4]. Low-angle sub-boundaries of these fragments were less effective barrier for the martensitic crystals to grow as compared to high-angle boundaries of grains. However, as accumulation of misorientated fragments occurs during repetitive γ-α-γ cycles (Figure 1), completeness of the direct transformation decreases. Reduction of the size of austenitic grain in melt-spun ribbons results in more considerable stabilization of reverted austenite as compared to the bulk alloy of the same chemical composition. It was found that after 30 γ-α-γ cycles of reverse diffussionless transformations in the ribbon, the amount of martensite on a free side dropped by 61%, on a contact side by 43%, while in a bulk alloy, only by 8% (Figure 2). Investigations indicated that the size effect of martensitic transformation yielded a higher degree of stabilization of reverted austenite on a free side of the ribbon as compared to a contact side.

**Figure 3:**
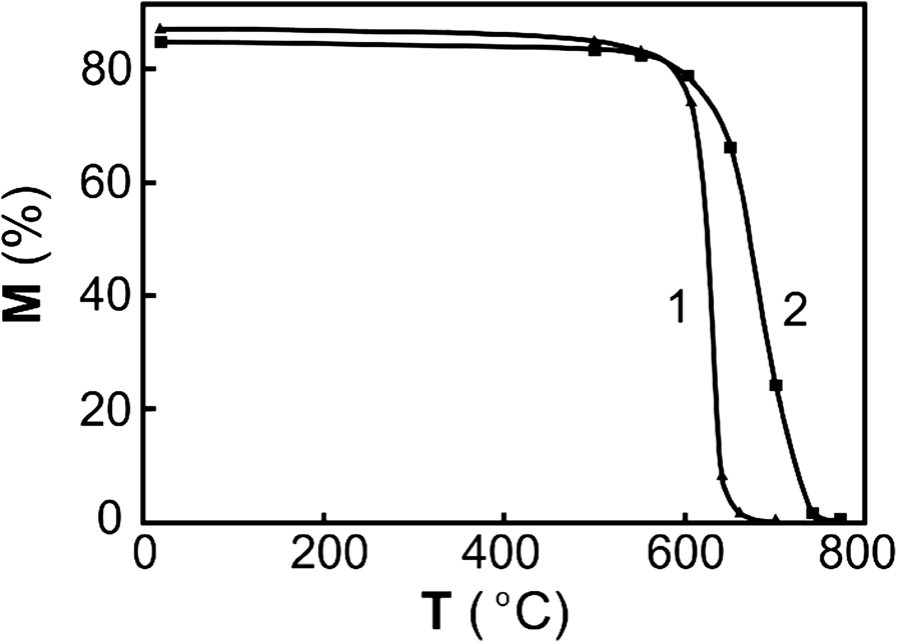
**Amount of martensite vs heating temperature for reverse α–γ transformation.** Amount of martensite vs heating temperature for reverse α-γ transformation of quenched Fe-30.6 wt.% Ni-0.05 wt.% C alloy: 1 ribbon, 2 bulk alloy. It should be noted that an interval of α-γ transformation of the ribbon and the bulk alloy shifted to higher temperatures after γ-α-γ cycling. Temperature shift for the ribbon was about 80 K according to X-ray data (Figure 2). After repeated γ-α-γ cycles finish, temperature (*A*_k_) of reverse α-γ transformation remarkably increases (by 75 to 80 K after the 30 cycles of γ-α-γ transformations). Abovementioned results mean that it is necessary to heat a pre-quenched ribbon for each α-γ transformation to higher *А*_к_ temperature for the achievement of higher degree of phase hardening by γ-α-γ transformations. It should be noted that overheating of quenched alloy to the temperatures higher than *А*_к_ by 1015 K results in development of relaxation processes and to the corresponding decline the phase hardening.

It should be noted that an interval of α-γ transformation of the ribbon and the bulk alloy shifted to higher temperatures after γ-α-γ cycling. Temperature shift for the ribbon was about 80 K according to X-ray data (Figure 3). After repeated γ-α-γ cycles finish, temperature (*A*_k_) of reverse α-γ transformation remarkably increases (by 75 to 80 K after the 30 cycles of γ-α-γ transformations). Abovementioned results mean that it is necessary to heat a pre-quenched ribbon for each α-γ transformation to higher *А*_к_ temperature for the achievement of higher degree of phase hardening by γ-α-γ transformations. It should be noted that overheating of quenched alloy to the temperatures higher than *А*_к_ by 10 to 15 K results in development of relaxation processes and to the corresponding decline the phase hardening.

After quenching from melt, an initial austenitic structure was kept in the ribbon. The X-ray investigations showed an austenitic phase on the free side of the ribbon had been formed with the growth (100)_γ_ texture, typically for f.c.c. structure. This texture appeared occasionally on a contact surface. The degree of texturing notably changes throughout the ribbon in accordance with the change of cooling speed in the process of ribbon crystallization. Metallography analysis discovered a considerable inhomogeneity of ribbon microstructure: roughness of surface, difference of microstructure between the edges and center of a ribbon, appearance of gas cavities on a contact surface and presence of cuts and grooves on a free surface.

The features of direct contact zone (DCZ) between a ribbon and a disk-cooler for melt spinning during fabrication were reflected on contact and free surfaces. Considerable roughness of the surface can be associated with inhomogeneity of the flow of molten metal and high cooling speed.

Grains on the contact surface formed in DCZ had the shape extended toward the disk motion. Ratio of length of the grain to its width (ellipseness) was within 1.8 to 3.5. In the central part of the ribbon, such a ratio was higher compared to its periphery. The elongated form of grains was characteristic for a contact surface only and not observed inside and on free surface of the ribbon.

Inside the ribbon, these grains had a columnar form and were inclined toward the disk-cooler motion. The relaxation processes was found to progress in this material as a result of the gradual heating of disk surface. Practically, complete absence of residual stresses in a ribbon can be explained by relaxation processes. It means that in the process of crystallization, the low-temperature (below 0.25 *Т*_melt_) relaxation occurs, while the heating of disk results in the middle-temperature (below 0.25 to 0.5 *Т*_melt_) relaxation [11].

Triple junctions of grains with corner between boundaries about 120° were observed on both surfaces and inside the ribbon. According to [11], formation of 120° boundaries is a signature of collective recrystallization. The number of 120° boundaries increased in the last portions of the ribbon as a result of increasing of disk temperature during melt spinning with the size of grains increased. Nevertheless, the type of grains distribution by sizes was kept.

Quenching in liquid nitrogen caused essential difference of grain structure on both the sides of the ribbon as well as the gradient distribution of the amount of martensitic phase inside it. Austenitic grains on a contact surface was smaller than on a free surface. As a result of the size effect of the transformation, amounts of martensitic phase on a contact and free surfaces were different - 59% and 82%, respectively (Figure 2).

## Conclusions

Sharp heterogeneity of grain structure of melt-spun ribbons fabricated from iron-nickel alloys on a contact, free surfaces and inside the ribbons with pronounced gradiental distribution of volume part of the martensitic phase are observed after quenching. The amount of martensite in local areas of the ribbon was found to decrease as the number of nanocrystalline grains where γ-α transformation has not realized increased.

Reverse α-γ transformation of a diffusive character, occurring on a slow heating of the ribbon pre-quenched with formation of martensite, resulted in a considerable grain refining of austenite formed. Austenite appeared to be a completely stabilized in relation to the next direct martensitic transformation as a result of presence of a significant quantity of ultra-dispersive and nanocrystalline components of reverted austenite after single γ-α-γ transformations.

## Abbreviations

DCZ: direct contact zone
f.c.c.: face centered cubic
